# Hospital Construction and Organization

**Published:** 1876-01

**Authors:** 


Hospital Construction and Organization. The Johns Hopkins' Hospital, of Baltimore. William Wood & Co., New York. Book pp. 353.

This work comes from the hands of the press neatly bound and containing 353 pages. It contains five different letters, written by John S. Billings, M.D., Norton Folsom, M.D., Joseph Jones, M.D., Caspar Morris, M.D., and Stephen Smith, M.D., presented to the trustees of the Johns Hopkins' Hospital, on the best mode of constructing, ventilating, and conducting in such a way as will be most conducive to the interest of the sick. The different letters, coming from gentlemen of such prominence in the profession, written with such precision and illustrated with numerous diagrams, make the work one of special interest to the profession and suffering humanity at large.

